# Plasma Proteomics Reveals Biomarkers and Undulating Changes in Metabolic Aging

**DOI:** 10.34133/research.1004

**Published:** 2025-12-04

**Authors:** Jijuan Zhang, Hancheng Yu, Yurong Xiong, Dan Xue, Shuang Chen, Juanjuan Li, Xianli Li, Jinchi Xie, Yuxiang Wang, Kun Xu, Gang Liu, Yunfei Liao, An Pan, Tingting Geng

**Affiliations:** ^1^ Department of Epidemiology and Biostatistics, Ministry of Education Key Laboratory of Environment and Health, School of Public Health, Tongji Medical College, Huazhong University of Science and Technology, Wuhan, Hubei Province, China.; ^2^ Department of Nutrition and Food Hygiene, Hubei Key Laboratory of Food Nutrition and Safety, School of Public Health, Tongji Medical College, Huazhong University of Science and Technology, Wuhan, Hubei Province, China.; ^3^ Centre for Obesity and Diabetes Research, School of Public Health, Tongji Medical College, Huazhong University of Science and Technology, Wuhan, Hubei Province, China.; ^4^ Department of Endocrinology, Wuhan Union Hospital, Tongji Medical College, Huazhong University of Science and Technology, Wuhan, Hubei Province, China.

## Abstract

Large-scale proteomics enables the identification of biomarkers and undulations in metabolic aging. This study aimed to develop a metabolic age (MA) and identify proteomic biomarkers and their undulating changes during metabolic aging. Using UK Biobank data, MA was developed from mortality-associated metabolomic profiles (nuclear magnetic resonance platform) in 203,491 participants. Associations between 2,923 plasma proteins (Olink Explore 3072 platform) and metabolic aging phenotypes, including MA, telomere length, frailty index, incident type 2 diabetes, cardiovascular disease, and mortality, were examined in 24,920 participants via Cox proportional hazards or linear models. Differential expression–sliding window analysis captured protein waves during metabolic aging in 7,092 participants. MA improved the predictions of mortality, cardiovascular disease, and type 2 diabetes beyond conventional risk factors (*C*-index up to 0.786) and correlated strongly with chronological age (Spearman’s *r*: 0.876). Sixty proteins were associated with all metabolic aging phenotypes. Among them, growth differentiation factor 15 (GDF15), urokinase plasminogen activator surface receptor (PLAUR), tumor necrosis factor receptor superfamily member 10A (TNFRSF10A), tumor necrosis factor receptor superfamily member 10B (TNFRSF10B), gamma-interferon-inducible lysosomal thiol reductase (IFI30), hepatocyte growth factor (HGF), WAP 4-disulfide core domain protein 2 (WFDC2), collagen alpha-3(VI) chain (COL6A3), polymeric immunoglobulin receptor (PIGR), insulin-like growth factor-binding protein 4 (IGFBP4), and tumor necrosis factor receptor superfamily member 27 (EDA2R) ranked within the top 20 for at least 4 phenotypes based on *P* values. Pathway analysis highlighted symbiont entry into host cell and cytokine–cytokine receptor interaction in metabolic aging. Proteins showed undulating changes during metabolic aging, with 3 peaks at 44, 51, and 63 years. MA–protein trajectories clustered into 3 groups. Groups 1 and 3 exhibited linear increases with MA, whereas group 2 showed nonlinear increases. In conclusion, the identification of plasma proteomic biomarkers and their undulating changes in metabolic aging provides a critical foundation for developing clinical markers and precision interventions to prevent accelerated metabolic aging.

## Introduction

The global population is aging rapidly, with an estimated 808.9 million individuals aged 65 and older in 2023, and is forecast to rise to 2.2 billion by the late 2070s [1,2]. Metabolic function progressively declines with advancing age [3], and accelerated metabolic aging markedly elevates the risk of cardiometabolic diseases and mortality [4–7]. Consequently, mitigating accelerated metabolic aging is critical for preventing cardiometabolic diseases and promoting healthy aging.

Metabolic aging is a multifaceted process manifesting across molecular, cellular, and organismal levels. For example, at the molecular level, dysregulated metabolites signal accelerated metabolic aging. Metabolic age (MA), derived from metabolomic profiles, can capture aging rate variability and quantify metabolic age acceleration (MAA) more accurately than chronological age [8,9]. At the cellular level, markers such as telomere attrition may reflect accelerated metabolic aging, while at the organismal level, it is associated with frailty, elevated cardiometabolic disease risks, and increased mortality [3,10,11]. Integrating these multidimensional indicators achieves a more comprehensive assessment of metabolic aging.

Plasma proteins, products of gene expression, serve as key upstream regulators of metabolic function. Although previous studies have linked genes like APOB and CDKN2A/B to an increased risk of cardiometabolic diseases [12], the relationship between plasma proteins and metabolic aging remains largely unexplored. Systematic identification of proteins associated with metabolic aging can uncover biomarkers and underlying biological pathways of metabolic aging. Additionally, while many plasma proteins have been shown to change with chronological age [13,14], little is known about their dynamic changes during metabolic aging. Exploring undulating protein changes during metabolic aging may reveal critical intervention windows to mitigate accelerated metabolic aging.

**Fig. 1.**
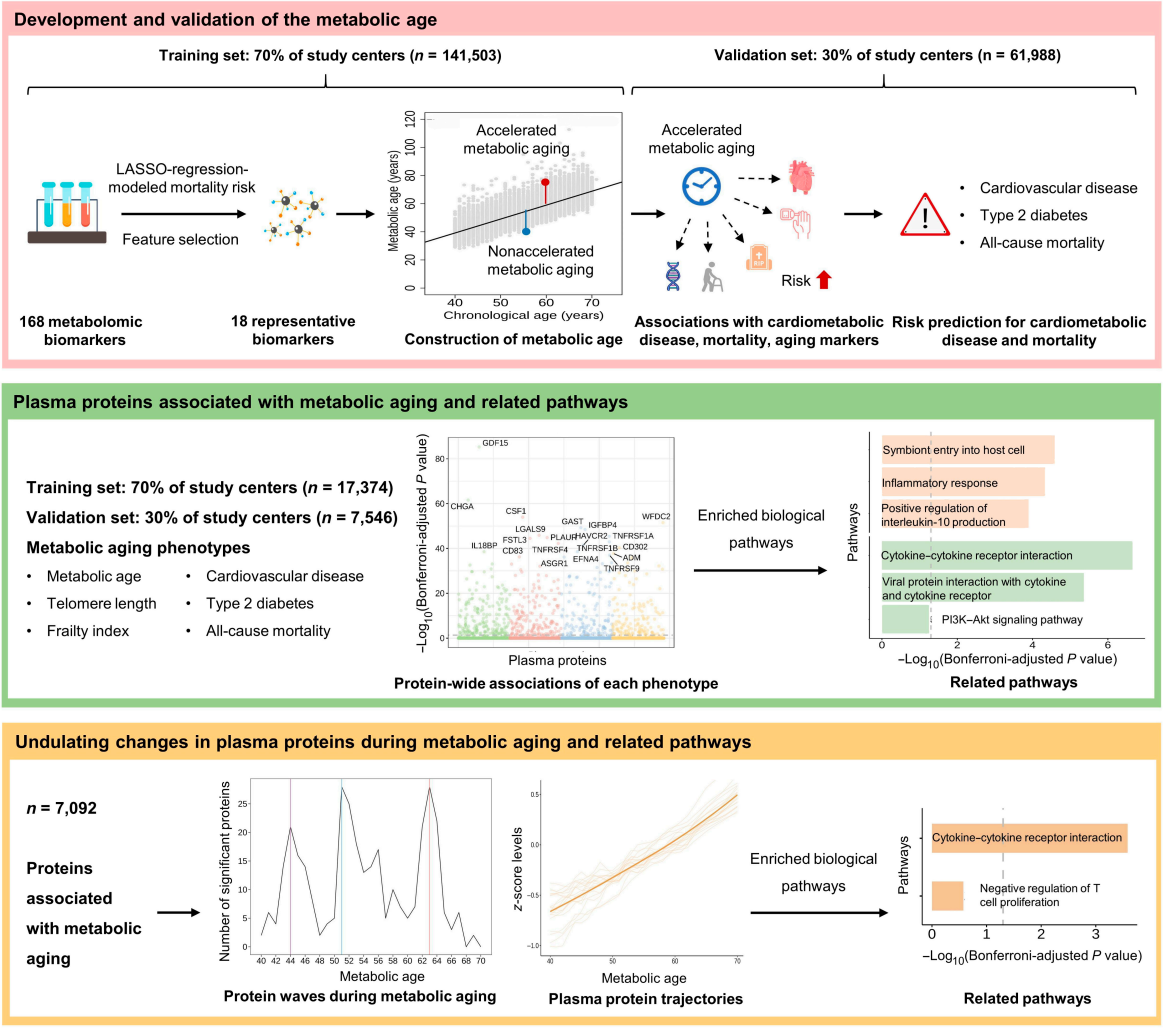
Study overview. LASSO, least absolute shrinkage and selection operator; PI3K–Akt, phosphoinositide 3-kinase–protein kinase B.

In this study, we conducted a large-scale metabolomic and proteomic analysis to (a) develop and validate an MA, (b) identify plasma proteins associated with metabolic aging phenotypes (including MA, telomere length, cardiometabolic disease risk, frailty, and mortality) and related biological pathways, and (c) explore undulating protein changes during metabolic aging and related biological pathways (Fig. 1).

## Results

### Characteristics of participants

This study included 203,491 participants (44.0% men; mean (standard deviation) age: 56.0 (8.1) years) with baseline metabolomic data for the development and validation of MA. Among the 203,491 participants, over median follow-ups of 13.0 to 13.9 years, there were 19,133 incident cases of cardiovascular disease (CVD), 7,021 incident cases of type 2 diabetes (T2D), and 14,207 deaths. Additionally, 24,920 participants were included to investigate proteome-wide associations with CVD, T2D, mortality, MA, frailty index (FI), and telomere length. Furthermore, 7,092 participants were included to evaluate undulating protein changes during metabolic aging (Table S1 and Fig. S1). Baseline characteristics differed slightly between participants with and without metabolomic data, as well as between those with both metabolomic and proteomic data and others, with standardized mean differences ≤0.087 (Tables S2 and S3).

### Development and validation of MA

**Fig. 2.**
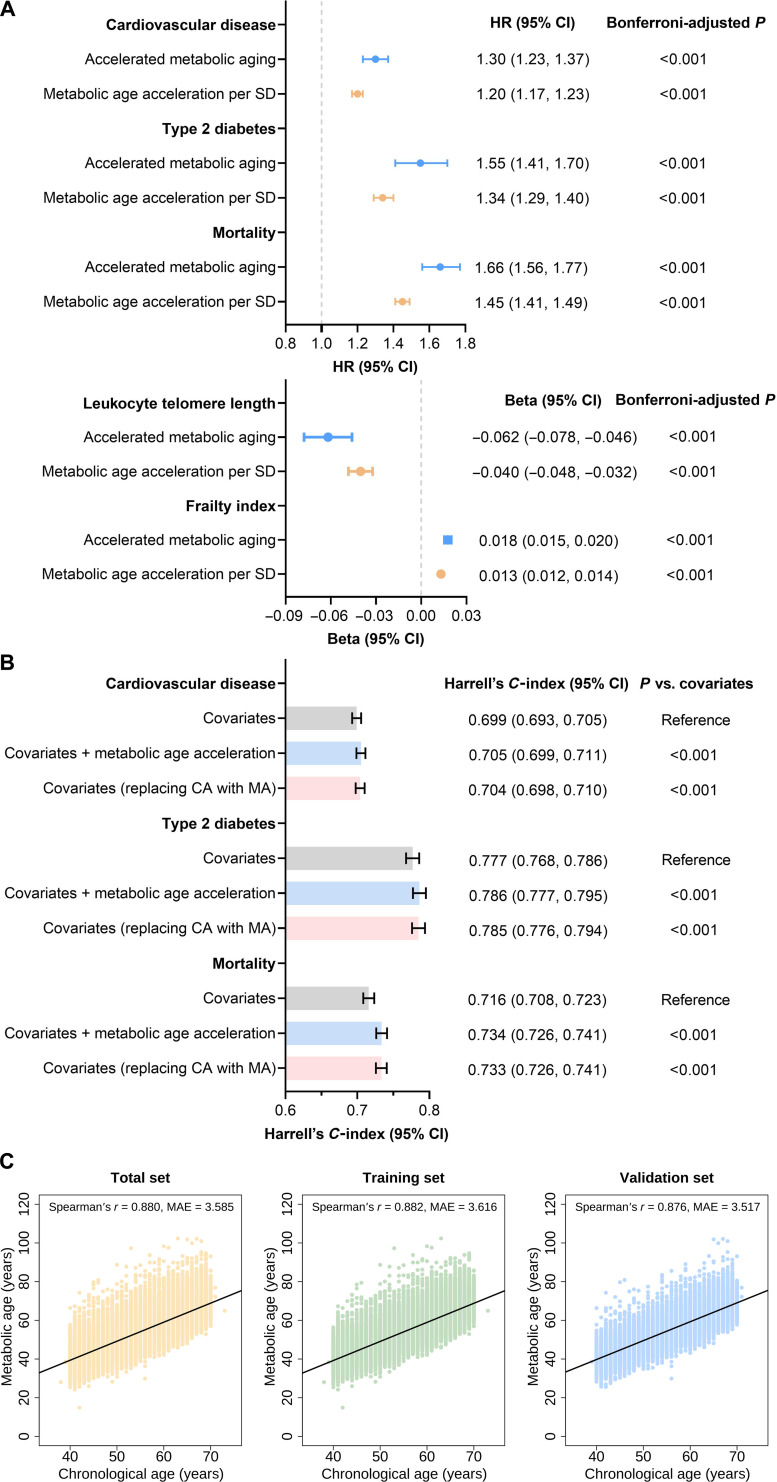
Development and validation of metabolic age. (A) Associations of accelerated metabolic aging with the risks of cardiometabolic disease and mortality, leukocyte telomere length, and frailty index in the validation set. (B) Predictive performance of metabolic age and metabolic age acceleration for cardiometabolic disease and mortality in the validation set. (C) Correlation between metabolic age and chronological age in the total set, the training set, and the validation set. In the top portion of the figure, the mean absolute error and Spearman’s correlation coefficient between metabolic age and chronological age are displayed. CA, chronological age; CI, confidence interval; HR, hazard ratio; MA, metabolic age; MAE, mean absolute error; SD, standard deviation.

Using the least absolute shrinkage and selection operator (LASSO) Cox model, 18 metabolomic indicators were selected to construct the MA (Text S1 and Table S4). Ranked by the absolute value of their LASSO coefficients, glycoprotein acetyls, degree of unsaturation of fatty acids, and average diameter for very-low-density lipoprotein particles were identified as more important indicators. In the validation set (Fig. 2), MA and MAA improved the predictions of mortality and cardiometabolic diseases (CVD and T2D) beyond traditional risk factors, with *C*-indices of up to 0.786 (all *P* values <0.001). Accelerated metabolic aging was related to higher risks of mortality and cardiometabolic diseases (CVD and T2D) (hazard ratio: 1.30 to 1.66), as well as a shorter telomere length (*β*: −0.062; 95% confidence interval [CI]: −0.078, −0.046) and a higher FI (*β*: 0.018; 95% CI: 0.015, 0.020). A strong correlation was observed between MA and chronological age (Spearman’s *r* = 0.876, mean absolute error = 3.517).

### Proteome-wide associations with metabolic aging

**Fig. 3.**
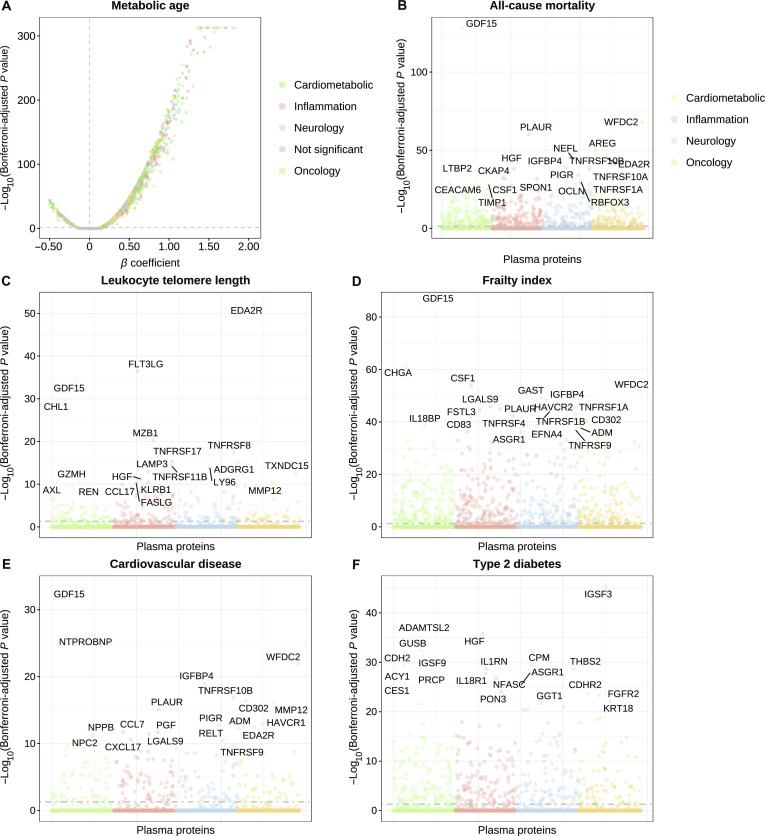
Associations of plasma proteins with (A) metabolic age, (B) all-cause mortality, (C) leukocyte telomere length, (D) frailty index, (E) cardiovascular disease, and (F) type 2 diabetes. Proteins labeled in the plots are the top 20 proteins ranked by Bonferroni-adjusted *P* values. The horizontal dashed line represents Bonferroni-adjusted *P* value = 0.05. In (A), gray dots represent nonsignificant proteins, while proteins from the cardiometabolic, neurology, inflammation, and oncology panels are highlighted in other distinct colors. In (B) to (F), proteins belonging to the cardiometabolic, neurology, inflammation, and oncology panels are shown in different colors.

**Fig. 4.**
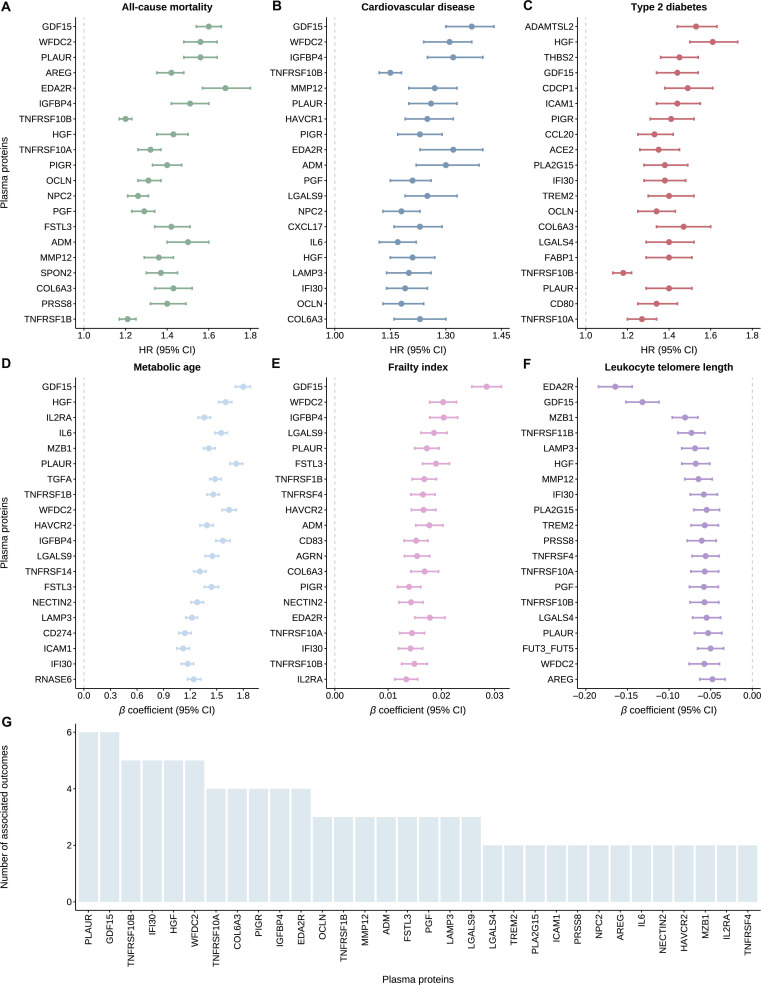
Top 20 metabolic-aging-related proteins ranked by Bonferroni-adjusted *P* values for (A) all-cause mortality, (B) cardiovascular disease, (C) type 2 diabetes, (D) metabolic age, (E) frailty index, and (F) leukocyte telomere length. (G) Top 20 metabolic-aging-related proteins for 2 or more metabolic aging phenotypes.

We assessed metabolic aging through multiple phenotypes including incident T2D and CVD, all-cause mortality, telomere length, FI, and MA. In the training set, 219 proteins were associated with CVD risk, 416 with T2D risk, 535 with all-cause mortality, 212 with telomere length, 626 with FI, and 1,924 with MA. Sixty proteins were associated with all 6 phenotypes and thus were regarded as metabolic-aging-related proteins (Fig. 3 and Table S5). Proteins associated with metabolic aging were primarily involved in symbiont entry into host cell, inflammatory response, positive regulation of interleukin-10 production, cytokine–cytokine receptor interaction, and viral protein interaction with cytokine and cytokine receptor (all Bonferroni-adjusted *P* <0.05, Fig. S2). Sensitivity analyses excluding incident cases of CVD, T2D, and deaths within the first year yielded consistent results (Table S6). Among the 60 proteins associated with metabolic aging, the top 20 proteins for each phenotype, ranked by Bonferroni-adjusted *P* values, are presented in Fig. 4A to F. The top 20 proteins were negatively associated with telomere length but positively associated with all other phenotypes. Notably, 11 core metabolic-aging-related proteins were highlighted: urokinase plasminogen activator surface receptor (PLAUR) and growth differentiation factor 15 (GDF15) ranked among the top 20 proteins across all 6 phenotypes; tumor necrosis factor receptor superfamily member 10B (TNFRSF10B), gamma-interferon-inducible lysosomal thiol reductase (IFI30), hepatocyte growth factor (HGF), and WAP 4-disulfide core domain protein 2 (WFDC2) across 5 phenotypes; and tumor necrosis factor receptor superfamily member 10A (TNFRSF10A), collagen alpha-3(VI) chain (COL6A3), polymeric immunoglobulin receptor (PIGR), insulin-like growth factor-binding protein 4 (IGFBP4), and tumor necrosis factor receptor superfamily member 27 (EDA2R) across 4 phenotypes (Fig. 4G).

In the validation set, 68 proteins were associated with CVD risk, 176 with T2D risk, 240 with all-cause mortality, 96 with telomere length, 400 with FI, and 1,692 with MA (Table S7). Among proteins associated with each metabolic aging phenotype in the training set, 25.1% to 86.2% were also associated with the corresponding phenotype in the validation set, with consistent directions of association. Notably, 99.1% to 99.9% showed consistent directions of association with those in the training set regardless of statistical significance (Table S8). Of the 60 proteins associated with all 6 metabolic aging phenotypes in the training set, 38 (63.3%) showed significant associations with 4 to 6 phenotypes in the validation set (Table S7). For the top 20 metabolic-aging-related proteins of each phenotype in the training set, 70% to 100% were also associated with the corresponding phenotype in the validation set, with consistent directions of association, and 100% showed consistent directions of association with those in the training set regardless of statistical significance (Table S9). Among the 11 key metabolic-aging-related proteins identified in the training set, GDF15, HGF, and TNFRSF10A were associated with all 6 phenotypes in the validation set; PLAUR, WFDC2, IFI30, TNFRSF10B, and PIGR with 5 phenotypes; COL6A3 and EDA2R with 4 phenotypes; and IGFBP4 with 3 phenotypes (Table S7).

### Undulating protein changes during metabolic aging

**Fig. 5.**
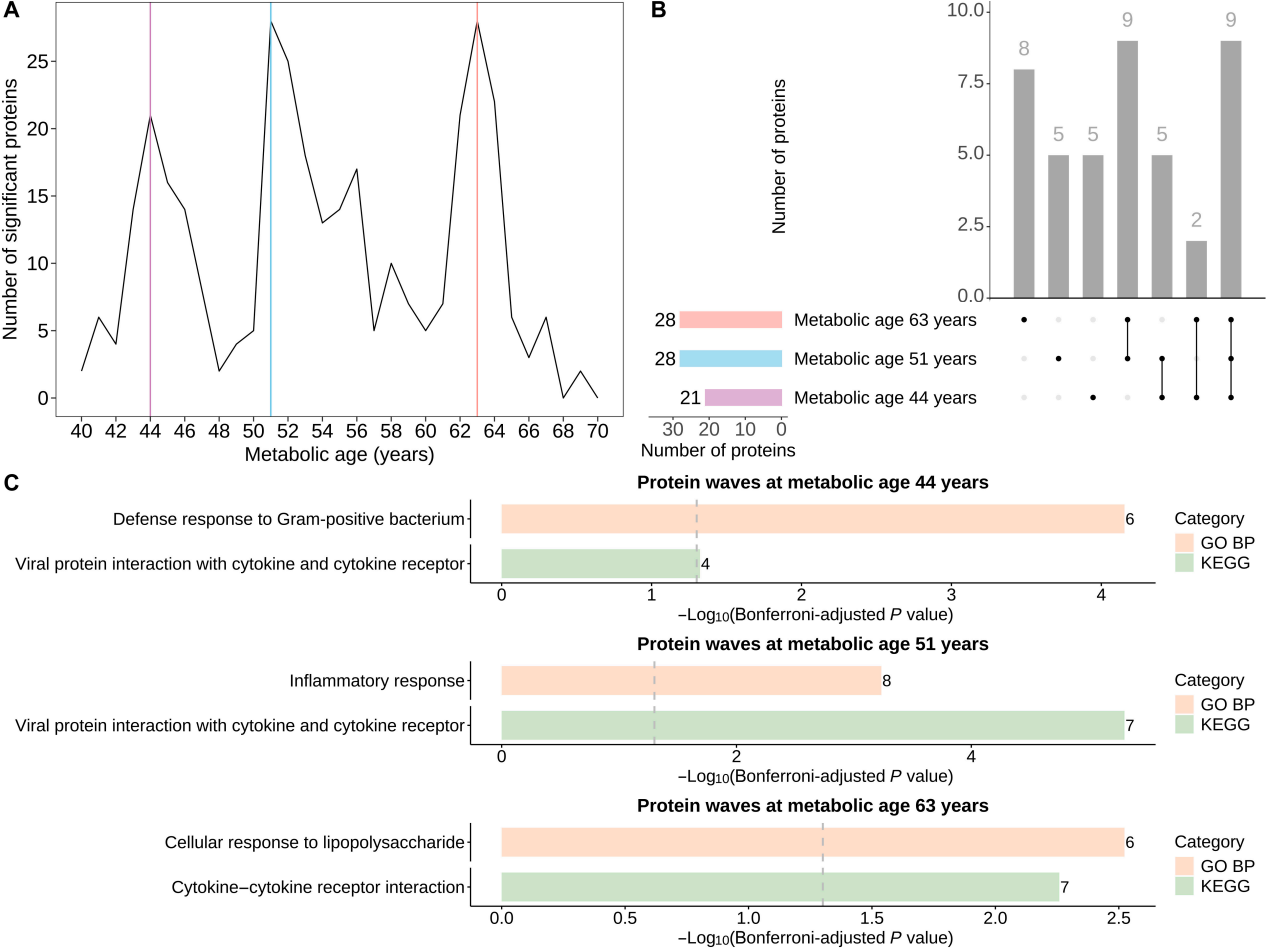
Undulating protein changes during metabolic aging. (A) Number of plasma proteins differentially expressed during metabolic aging. (B) Upset plot showing the shared and unique proteins differentially expressed at different peaks of metabolic age. (C) Top enriched pathways of proteins differentially expressed at different peaks of metabolic age. *P* values were calculated under 2-sided tests, and statistical significance was defined as a Bonferroni-adjusted *P* <0.05 (dashed vertical line). The number near each bar is the number of observed proteins in each pathway. GO BP, Gene Ontology—biological process; KEGG, Kyoto Encyclopedia of Genes and Genomes.

For metabolic-aging-related proteins, 3 peaks in differentially expressed proteins were observed at MAs 44, 51, and 63 years (Fig. 5A). These peaks persisted across different *P* value thresholds, underscoring the stability of protein waves during metabolic aging (Fig. S3). The largest numbers of differentially expressed proteins were observed at 51 and 63 years (28 proteins each), followed by 44 years (21 proteins). These peaks shared some overlapping proteins but also exhibited distinct differentially expressed proteins (Fig. 5B and Table S10). Nine proteins, namely, hepatitis A virus cellular receptor 2 (HAVCR2), interleukin-6 (IL6), galectin-9 (LGALS9), nectin-2 (NECTIN2), PLAUR, ribonuclease K6 (RNASE6), TNFRSF10B, tumor necrosis factor receptor superfamily member 1B (TNFRSF1B), and WFDC2, were consistently significant across all 3 peaks (Table S10). Proteins differentially expressed at 44 years were primarily associated with defense response to Gram-positive bacterium and viral protein interaction with cytokine and cytokine receptor, those differentially expressed at 51 years were primarily associated with inflammatory response and viral protein interaction with cytokine and cytokine receptor, while proteins differentially expressed at 63 years were mainly involved in cellular response to lipopolysaccharide and cytokine–cytokine receptor interaction (all Bonferroni-adjusted *P* <0.05, Fig. 5C). In sensitivity analyses, proteins with a false-discovery-rate-adjusted *P* <0.05 were considered statistically significant. At 44, 51, and 63 years, 39, 47, and 49 differentially expressed proteins were identified, respectively (Table S11), with enriched pathways largely consistent with the main analysis (Fig. S4).

**Fig. 6.**
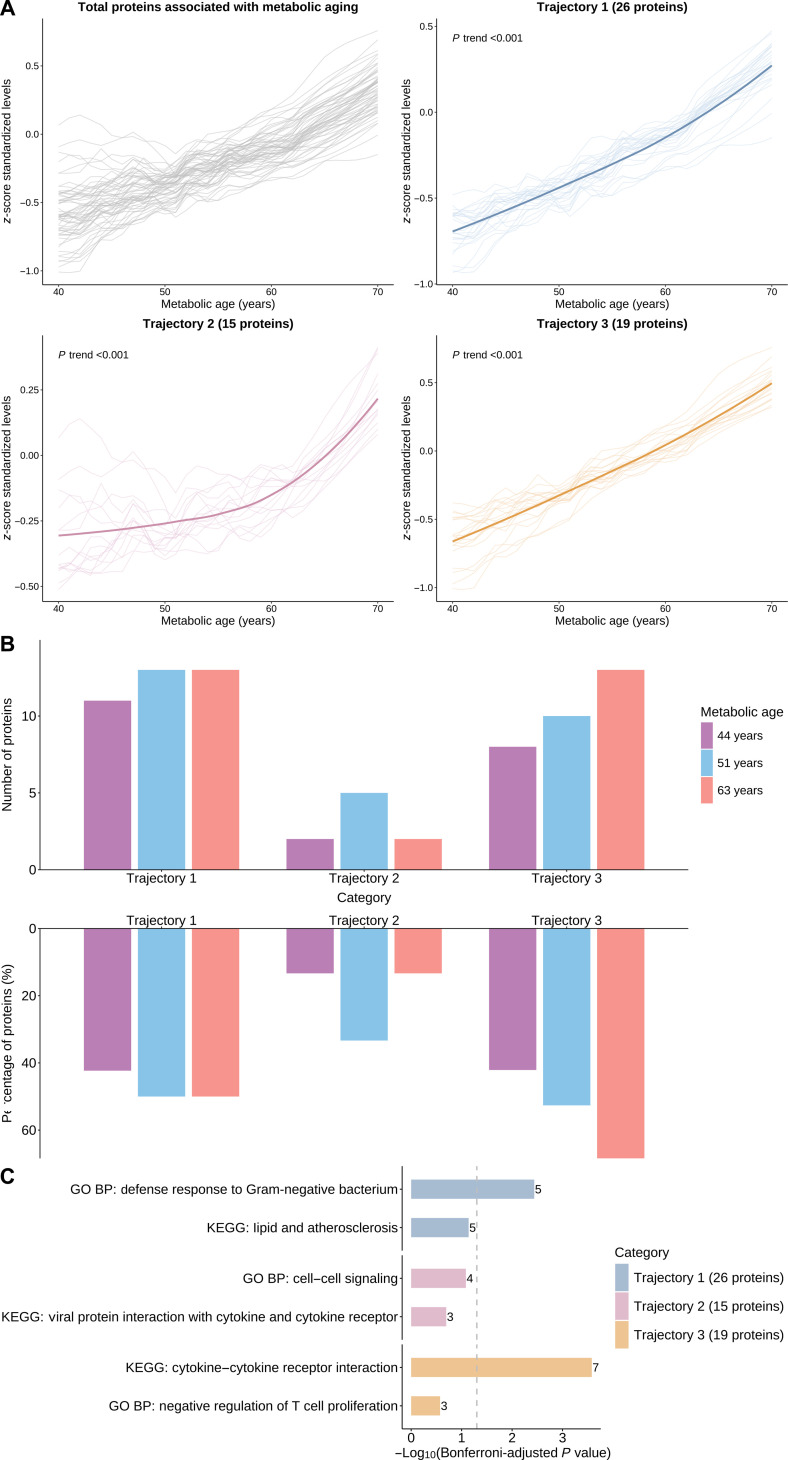
Plasma protein trajectories during metabolic aging. (A) Different trajectories of protein levels across metabolic age. Protein levels were *z*-score standardized, and their trajectories across metabolic age were modeled using locally weighted scatterplot smoothing models. For each cluster, the thick line represents the average trajectory. Mann–Kendall tests were applied to evaluate monotonic trends in the trajectories. (B) Differential expression of proteins across trajectory groups and metabolic age peaks. (C) Top enriched pathways of proteins in 3 trajectory groups. *P* values were calculated under 2-sided tests, and statistical significance was defined as a Bonferroni-adjusted *P* <0.05 (dashed vertical line). The number near each bar is the number of observed proteins in each pathway. GO BP, Gene Ontology—biological process; KEGG, Kyoto Encyclopedia of Genes and Genomes.

We categorized 60 metabolic-aging-related proteins into 3 groups based on their trajectories with MA: 26 proteins in group 1, 15 in group 2, and 19 in group 3 (Table S12). Proteins showed linear increases with MA in groups 1 and 3 and nonlinear increases in group 2 (all *P* trends <0.001, Fig. 6A). Proteins in group 1 exhibited comparable expression across all 3 peaks, those in group 2 had higher expression at 51 years than at 44 or 63 years, and protein expression in group 3 rose from 44 to 51 years and peaked at 63 years (Fig. 6B). Notably, proteins in group 1 were primarily involved in defense response to Gram-negative bacterium (Bonferroni-adjusted *P* = 0.004), group 2 in cell–cell signaling (Bonferroni-adjusted *P* = 0.082), and group 3 in cytokine–cytokine receptor interaction (Bonferroni-adjusted *P* <0.001, Fig. 6C).

## Discussion

In this study, we developed an MA based on plasma metabolomic profiles as one of metabolic aging phenotypes. Proteome-wide analyses identified 60 metabolic-aging-related proteins associated with MA, CVD, T2D, all-cause mortality, telomere length, and FI, of which 38 (63.3%) replicated across 4 to 6 metabolic aging phenotypes in the internal validation set. Among 60 metabolic-aging-related proteins, GDF15, PLAUR, TNFRSF10A, TNFRSF10B, IFI30, HGF, WFDC2, COL6A3, PIGR, IGFBP4, and EDA2R ranked within the top 20 for at least 4 phenotypes, indicating their central roles in metabolic aging. Pathway analysis highlighted symbiont entry into host cell and cytokine–cytokine receptor interaction for metabolic-aging-related proteins. Differential protein expression was observed at MAs 44, 51, and 63 years, suggesting these time points as critical milestones during metabolic aging. In addition, we identified 3 distinct patterns of MA–protein trajectories, revealing heterogeneity in protein dynamics during metabolic aging.

We identified 18 mortality-associated metabolomic indicators using a LASSO Cox model to construct the MA. A previous study with a similar approach reported 54 such indicators [15]. Several methodological differences may account for the discrepancy in the number of identified indicators. The previous study included a broader set of derived indicators (e.g., ratios and percentages) for screening, whereas we focused on nonderived indicators to reduce potential collinearity and overfitting. Differences in the *λ* penalty parameter of the LASSO Cox model and in sample size may also have contributed. Nonetheless, the overlap in identified metabolic indicators is substantial, with both studies identifying glycoprotein acetyls, the degree of unsaturation of fatty acids, and the average diameter of very-low-density lipoprotein particles as strongly associated with mortality.

Previous studies have constructed biological ages by predicting chronological age [5,7]. However, the closer these biological ages approximate chronological age, the less information independent of chronological age they provide for accurate assessment of health and prediction of health outcomes. This issue conflicts with the expectation that biological age should outperform chronological age in evaluating health status and forecasting future health risks [16]. To address this, we developed an MA from mortality-associated metabolomic profiles to quantify survival differences among individuals of the same chronological age. The MA effectively predicted mortality and incident cardiometabolic diseases. Accelerated metabolic aging, assessed by the MA, was related to higher risks of mortality and cardiometabolic diseases, as well as key aging hallmarks, including telomere attrition and increased frailty. Also, the MA showed strong correlation with chronological age. Taken together, these findings support the clinical utility of the MA for accurate assessment of metabolic aging and risk stratification.

Proteome-wide association analyses identified 60 proteins associated with MA, CVD, T2D, all-cause mortality, telomere length, and FI, thereby marking them as metabolic-aging-related proteins. Among 60 metabolic-aging-related proteins, GDF15, PLAUR, TNFRSF10A, TNFRSF10B, IFI30, HGF, WFDC2, COL6A3, PIGR, IGFBP4, and EDA2R ranked within the top 20 for at least 4 metabolic aging phenotypes based on Bonferroni-adjusted *P* values, highlighting their key roles in metabolic aging. GDF15 signals through the hindbrain GDNF family receptor α-like, modulating downstream molecules such as extracellular signal-regulated kinases and protein kinase B to mediate appetite suppression and metabolic homeostasis. These effects position GDF15 as a promising therapeutic target for obesity, nonalcoholic fatty liver disease, and diabetes [17,18]. Additionally, under stress or disease, circulating GDF15 levels may increase, indicating its potential as a biomarker and possibly representing a compensatory protective response to physiological adversity [17,18]. Circulating PLAUR is primarily produced through the enzymatic cleavage of its membrane-bound form on immune cells. Given its role in promoting chronic inflammation and atherosclerosis, it represents a promising immune-derived therapeutic target for atherosclerotic disease [19]. TNFRSF10A and TNFRSF10B, members of the tumor necrosis factor receptor superfamily, are membrane-bound TNF-related apoptosis-inducing ligand (TRAIL) receptors that primarily induce caspase-dependent apoptosis. They enter circulation mainly via ectodomain shedding and extracellular vesicles. It is speculated that elevated circulating TNFRSF10A and TNFRSF10B may sequester TRAIL, limiting its engagement with functional membrane receptors and attenuating its pro-apoptotic and anti-inflammatory effects [20,21]. Also, high levels of circulating TNFRSF10A and TNFRSF10B may be a biomarker of chronic inflammation and active apoptosis, which drive the development of cardiometabolic disease [20,21]. EDA2R, also a member of the tumor necrosis factor receptor superfamily, binds its ligand ectodysplasin A2 to activate the noncanonical nuclear factor-κB pathway, thereby regulating chronic inflammatory responses and related processes [22]. Multi-tissue transcriptomic analyses have linked increased EDA2R expression to aging across diverse tissues, suggesting EDA2R antagonists as potential antiaging therapeutic targets [23]. IFI30 is a lysosomal thiol reductase that reduces disulfide bonds in exogenous antigens, thereby facilitating antigen processing and presentation and contributing to immune and inflammatory responses. IFI30 genetic variants are associated with plasma C-reactive protein levels, hyperglycemia, and diabetes [24,25]. HGF binds to its receptor c-Met to activate pathways such as phosphoinositide 3-kinase–protein kinase B, thereby alleviating insulin resistance, enhancing glucose homeostasis, promoting angiogenesis, and inhibiting fibrosis [26,27]. Consistent with our findings, however, several studies have shown that elevated circulating HGF levels are positively associated with insulin resistance, myocardial infarction, and heart failure. These paradoxical associations may reflect a state of HGF resistance and support its potential as a diagnostic or prognostic biomarker for cardiometabolic diseases [26,28]. WFDC2 primarily inhibits serine protease- and matrix metalloproteinase-mediated degradation of type I collagen, thereby promoting fibrosis in multiple organs (e.g., heart and kidney) [29,30]. COL6A3, a key component of collagen VI in the extracellular matrix, can be cleaved to release endotrophin, a bioactive fragment that drives fibrosis, inflammation, and metabolic dysfunction [31]. PIGR is predominantly localized to the basolateral membrane of secretory epithelial cells, where it mediates the transcytosis of immunoglobulin A and immunoglobulin M to mucosal surfaces to generate secretory antibodies and maintain barrier immunity. Circulating PIGR, largely derived from cleaved secretory fragments, may contribute to immune regulation and serve as a biomarker of subclinical atherosclerosis and CVD [32,33]. IGFBP4 transports insulin-like growth factors (IGFs), binding them to regulate their bioavailability and activity. By modulating IGF-driven phosphoinositide 3-kinase–protein kinase B and mitogen-activated protein kinase–extracellular regulated protein kinase pathways that regulate cell proliferation, differentiation, and glucose–lipid homeostasis, it may indirectly influence metabolism and aging [34]. Collectively, these key proteins influence cardiometabolic health and aging through multiple biological processes, predominantly including inflammation and immune regulation, tissue remodeling and fibrosis, glucose–lipid metabolism, cell proliferation, and apoptosis.

During metabolic aging, plasma protein expression exhibited 3 prominent peaks at 44, 51, and 63 years. The earliest, at 44 years, was also reported in a previous plasma proteomic analysis [35]. More specifically, tissue-specific proteomics showed that protein expression in the aorta and pancreas also peaked around 44 years, suggesting coordinated alteration of vascular and metabolic organs at this age [36]. This midlife transition is characterized by visceral fat accumulation, muscle loss, insulin resistance, and endothelial dysfunction, which may accelerate aging and heighten cardiometabolic risks [37]. In addition, a proteomic study focusing on immune-related proteins reported peaks in serum autoantibody expression at 50 and 62 years [38], closely aligning with our observations and implicating immune alteration as a key contributor at these later transitions. Notably, 9 overlapping proteins, namely, HAVCR2, IL6, LGALS9, NECTIN2, PLAUR, RNASE6, TNFRSF10B, TNFRSF1B, and WFDC2, were consistently expressed across all 3 peaks. Most of them (HAVCR2, IL6, LGALS9, NECTIN2, PLAUR, RNASE6, TNFRSF10B, and TNFRSF1B) are primarily involved in immune and inflammatory regulation [19,39–42], underscoring the central role of these biological processes in metabolic aging. In addition, NECTIN2 is also a component of adherens junctions, maintaining tissue integrity and barrier function [43]. TNFRSF10B and TNFRSF1B, as tumor necrosis factor receptor family members, also regulate apoptosis and cell survival [39]. WFDC2 is mainly involved in fibrosis promotion [29,30]. Clinically, the identified peaks may represent critical milestones in metabolic aging and serve as time points for stage-specific screenings, for example, vascular and metabolic assessments in early midlife and immune function evaluations from midlife to late life. Individuals identified at elevated risk could then receive tailored interventions, such as lifestyle management (e.g., diet, exercise, or weight control) and appropriate prophylactic pharmacological interventions (e.g., lipid-lowering agents or anti-inflammatory therapies), to mitigate accelerated metabolic aging and reduce cardiometabolic disease risk.

Proteins associated with metabolic aging were classified into 3 groups based on their trajectories with MA. Proteins in different groups were specifically involved in characteristic biological pathways. These findings indicated heterogeneity in protein dynamics during metabolic aging and provided a basis for precise interventions on aging-related proteins. It is worth noting that the undulating protein changes and the MA–protein trajectories may be influenced by factors such as medication use and comorbidities [44], which should be carefully considered in relevant research. The biological significance of our findings remains to be confirmed, and future studies will be necessary to validate and extend our observations.

This study conducted a large-scale metabolomic and proteomic analysis to explore proteomic biomarkers and undulating changes in metabolic aging. The prospective design, the substantial number of measured metabolomic profiles and proteins, and the large sample size strengthen the research quality and the robustness of the findings. However, certain limitations warrant attention. First of all, while the Olink platform was used to quantify plasma proteins, it does not encompass the entire human proteome. Therefore, studies utilizing alternative proteomic platforms (e.g., SomaScan) are needed to complement our findings. Secondly, since this study predominantly included middle-aged and older White participants, undulating protein changes across other age spectrums remain underexplored, and the generalizability of results to other ethnic groups may be constrained. Thirdly, the MA and proteomic biomarkers has not been validated externally. Nevertheless, internal validation yielded robust results. Future studies with large-scale, multi-omics, and longitudinal data are warranted to confirm our findings and facilitate their translation into clinical practice. Fourthly, participants with metabolomic or proteomic data represented a subset of the original cohort, which may introduce potential selection bias. Nevertheless, baseline characteristic differences between those with and without metabolomic data, as well as between participants with both metabolomic and proteomic data and others, were minimal (standardized mean differences ≤0.087). Finally, given the small number of proteins in the pathway enrichment analyses of differentially expressed proteins and protein trajectory groups, the identified pathways should be interpreted with caution and warrant future validation.

In conclusion, this large-scale cohort study developed an MA as one of metabolic aging phenotypes and highlighted 11 central proteins for metabolic aging: GDF15, PLAUR, TNFRSF10A, TNFRSF10B, IFI30, HGF, WFDC2, COL6A3, PIGR, IGFBP4, and EDA2R. Pathway analysis underscored the critical roles of symbiont entry into host cell and cytokine–cytokine receptor interaction in metabolic aging. Notably, this study pinpointed 44, 51, and 63 years as critical milestones during metabolic aging and identified 3 protein groups with distinct MA–protein trajectories. Collectively, these findings provide a critical foundation for developing clinical markers and precision interventions to prevent accelerated metabolic aging.

## Materials and Methods

### Study population

The UK Biobank is a prospective cohort study that included over 500,000 individuals aged 40 to 69 years between 2006 and 2010. Further details regarding the UK Biobank are available elsewhere [45]. The UK Biobank was approved by the North West Multi-centre Research Ethics Committee (REC reference numbers 11/NW/0382, 16/NW/0274, and 21/NW/0157), and all participants provided written informed consent.

In this study, to construct and validate the MA, we included participants with baseline metabolomic, telomere, and FI data, excluding those with prevalent CVD or T2D at baseline, as well as those with baseline proteomic data to avoid sample overlap with proteome-wide association analyses. The final sample included 203,491 individuals from 21 assessment centers. We randomly selected 70% of the centers (15 centers; *n* = 141,503) as the training set, while the remaining 30% (6 centers; *n* = 61,988) comprised the validation set. For proteome-wide association analyses of metabolic aging, we included 24,920 participants with baseline metabolomic and proteomic data, no prevalent CVD or T2D, and complete baseline telomere and FI data. Participants were allocated to a training set (15 randomly selected centers, 70%; *n* = 17,374) and a validation set (6 remaining centers, 30%; *n* = 7,546). For exploring undulating protein changes during metabolic aging, 7,092 participants with baseline metabolomic and proteomic data; free of prevalent CVD, T2D, hypertension, and dyslipidemia; and not using antihypertensive, antidiabetic, or lipid-lowering medications at baseline were included. Figure S1 illustrates the study flow chart. The assessment centers included in the training and validation sets are listed in Table S13.

### Plasma metabolomics

Plasma metabolomic profiles from ~275,000 baseline participants were quantified using a high-throughput nuclear magnetic resonance platform, with detailed sample collection and metabolomics procedures described in a previous publication [46]. For this study, we analyzed 168 nonderived metabolomic biomarkers, encompassing fatty acids and their compositions, glycolysis metabolites, amino acids, ketone bodies, and lipoprotein lipids across 14 subclasses (Table S14). Prior to analysis, missing metabolomic values were imputed using the mean, and all metabolomic biomarkers were ln(*x* + 1) transformed and subsequently standardized to *z*-scores.

### Development and validation of the MA

We randomly selected 70% of the assessment centers (15 centers; *n* = 141,503) as the training set, while the remaining 30% (6 centers; *n* = 61,988) comprised the validation set. In the training set, a Cox model with LASSO penalty was used to select metabolomic indicators associated with mortality risk, with the penalty applied only to metabolomic indicators and not to covariates [4]. Ten-fold cross-validation and the “1 standard error rule” were applied to select the *λ* penalty parameter. A parametric proportional hazards model based on the Gompertz distribution was used to estimate MA from mortality-associated metabolomic indicators, representing the age at which a participant’s predicted mortality score aligns with the average mortality hazard [47]. The formula for calculating MA is provided in Text S1. MAA was determined by the residual from regressing MA on chronological age. A residual >0 indicates accelerated metabolic aging (metabolically older), while ≤0 indicates nonaccelerated metabolic aging (metabolically younger). In the validation set, MA was evaluated through (a) predictive performance of MA and MAA for mortality and cardiometabolic diseases (CVD and T2D); (b) associations of MAA with mortality, cardiometabolic diseases (CVD and T2D), and general aging markers (telomere length and FI); and (c) the Spearman’s correlation coefficient and mean absolute error between MA and chronological age.

### Plasma proteomics

The UK Biobank Pharma Proteomics Project is a consortium that profiled the plasma proteomes of 54,219 participants from the UK Biobank [48]. Details regarding the proteomics assay, data processing, and quality control procedures are available elsewhere [48]. In brief, protein profiling was performed using the Olink Explore 3072 platform, measuring 2,941 analytes corresponding to 2,923 proteins across 8 panels: cardiometabolic, cardiometabolic II, inflammation, inflammation II, neurology, neurology II, oncology, and oncology II. The proteomic data were expressed as normalized protein expression values. We used baseline proteomic data, excluding proteins with more than 30% missing values, which left us with an analytical set of 2,920 proteins (Table S15). Prior to analysis, missing normalized protein expression values were imputed using the mean, and all proteomic measures were standardized to *z*-scores.

### Ascertainment of cardiometabolic diseases and mortality

Cardiometabolic diseases included CVD and T2D. The first occurrences of cardiometabolic diseases were identified by mapping hospital admission, death register, primary care, and self-reported data. Diseases were defined by the International Classification of Diseases Tenth Edition codes: I20 to I25, I60 to I64, and I50 for CVD and E11 for T2D. All-cause mortality was identified via record linkage with national death registries. Person-years were calculated from baseline until the earliest of initial disease diagnosis, death, loss to follow-up, or censoring (2022 April 1).

### Assessment of frailty and leukocyte telomere length

At baseline, frailty was assessed using a 49-item FI, evaluating health deficits such as disease histories, functional status, self-rated health, depression, and pain [49]. Participants with ≥10 missing items were excluded. The FI (range 0 to 1) was calculated as the proportion of observed health deficits among 49 items, with higher scores indicating greater frailty. Baseline leukocyte telomere length was quantified via quantitative polymerase chain reaction and expressed as the ratio of telomere repeat copy number to single-copy gene number [50]. Telomere length values were technically adjusted, log-transformed, and standardized to *z*-scores.

### Statistical analysis

Cox proportional hazards models with multivariable adjustment were used to estimate hazard ratios and 95% CIs for associations of accelerated metabolic aging, MA, MAA, and protein levels with risks of cardiometabolic diseases and mortality. The proportional hazards assumption was evaluated using Schoenfeld residuals, with no evident violations detected. Multivariable linear regression models were used to estimate *β*s and 95% CIs for (a) associations of accelerated metabolic aging and MAA with FI and telomere length and (b) associations of protein levels with MA, FI, and telomere length. Models were adjusted for baseline covariates (Text S1), including age (years; continuous), sex (men, women), self-reported race (White, non-White), Townsend deprivation index (continuous), educational attainment (college or university degree, below college), body mass index (kg/m^2^; <25, 25 to 29.9, and ≥30), healthy diet (yes, no), alcohol consumption (current, noncurrent), physical activity (adequate, inadequate), smoking status (current, noncurrent), use of antihypertensive medications (yes, no) and lipid-lowering medications (yes, no), estimated glomerular filtration rate (ml/min/1.73 m^2^; continuous), and histories of hypertension (yes, no) and dyslipidemia (yes, no). We performed a sensitivity analysis excluding incident cases of cardiometabolic diseases or deaths within the first year to minimize potential reverse causation bias.

We employed the differential expression–sliding window analysis to capture plasma protein waves during metabolic aging [14]. MA ranged from 40 to 70 years, and 31 MA centers were selected, each allowing a ±1.5-year window. Differentially expressed proteins were identified using the linear regression model:

$$\text{Protein levels}=\alpha +\beta_{1}\times {\mathrm{MA}}_{\mathrm{low}/\text{high}}+\beta_{x}\times x+\varepsilon$$ (1)

where *α* is the intercept, *ε* is the residual error, *β*s represent regression coefficients, and *x* denotes the adjusted covariates, including age, sex, self-reported race, assessment center of participants, Townsend deprivation index, educational attainment, body mass index, healthy diet, alcohol consumption, physical activity, smoking status, and estimated glomerular filtration rate. At each MA center, proteins meeting a Bonferroni-corrected *P* value <0.05 were considered statistically significant. An illustrative example of the differential expression–sliding window analysis can be found in Text S1.

We applied a locally estimated scatterplot smoothing (LOESS) model to fit protein trajectories across MA. Mann–Kendall tests were applied to evaluate the presence of monotonic trends in the trajectories. Pairwise differences between LOESS estimates were assessed by the Euclidian distance, and hierarchical clustering using Ward’s minimum variance method was used to categorize the MA–protein trajectory patterns.

To explore the biological relevance of proteins, pathway enrichment analyses were conducted based on Gene Ontology—biological process and Kyoto Encyclopedia of Genes and Genomes terms.

Missing values of covariates were imputed using median values (continuous variables) and mode values (categorical variables) if the missing rate was ≤5%. When the missing rate was >5%, a missing indicator was applied [51].

Statistical analyses were performed utilizing the R software (version 4.1.3). All *P* values were 2-tailed, with a significance level at 0.05. To account for multiple comparisons, the Bonferroni correction was applied to adjust for *P* values.

## Data Availability

Data for this study were obtained from the UK Biobank (https://www.ukbiobank.ac.uk/), an open-access resource, under approved application number 88159.
