# Supplementary material for: Plasma Proteomics Reveals Biomarkers and Undulating Changes in Metabolic Aging
Source: Research (Wash D C). 2025 Dec 4;8:1004. doi: 10.34133/research.1004 (PMC12678049; doi:10.34133/research.1004)
Supplement: Supplementary 1 — Text S1 Figs. S1 to S4 Tables S1 to S15 [file research.1004.f1.zip › Supplementary methods and figures.docx]

**Jijuan Zhang, et al, Plasma proteomics reveals biomarkers and undulating changes in metabolic aging**

**Text S1.** Calculation of metabolic age, assessment of covariates and differential expression - sliding window analysis

**Fig. S1.** Study flow chart

**Fig. S2.** Top three enriched pathways of proteins associated with metabolic aging

**Fig. S3.** Plasma protein waves during metabolic aging under different thresholds of *P* values

**Fig. S4.** Top three enriched pathways of FDR-significant (*P* <0.05) proteins at different peaks of metabolic age

**Text S1.** Calculation of metabolic age, assessment of covariates and differential expression - sliding window analysis

**Calculation of metabolic age**

Referring to the method proposed by Levine et al. [1], a parametric proportional hazards model based on the Gompertz distribution was used to estimate metabolic age from mortality-associated metabolites. The following formula was used to calculate metabolic age, with the levels of each metabolite standardized as z-scores (mean of 0 and standard deviation of 1):

$$\text{Metabolic age = 136.8892 + }\frac{\text{ln[-0.0102 × }\frac{\text{-2.4788 × exp(xb)}}{\text{0.0104}}\text{]}}{\text{0.1002}}$$

$$\text{xb}\text{ = -14.51855835 - 0.10024841 × Average Diameter for VLDL Particles }$$

$$\text{- 0.24881753 × Degree of Unsaturation - 0.10422540 × Omega-3 Fatty Acids }$$

$$\text{- 0.25733988 × Linoleic Acid - 0.01934856 × Alanine}$$

$$\text{- 0.02969278 × Leucine - 0.12667245 × Valine + 0.04495469 × Phenylalanine }$$

$$\text{+ 0.04476189 × Tyrosine + 0.05750399 × Lactate + 0.06882400 × Acetate}$$

$$\text{+ 0.04701745 × Acetoacetate + 0.04832028 × Acetone - 0.05266166 × Albumin}$$

$$\text{+ 0.31846241 × Glycoprotein Acetyls + 0.13376079 × Free Cholesterol in Very Small VLDL}$$

$$\text{+ 0.04626309 × Free Cholesterol in Very Large HDL }$$

$$\text{- 0.05699244 × Cholesteryl Esters in Small HDL }$$

$$\text{+ 0.09756780 × Chronological Age (years)}$$

**Assessment of covariates**

At baseline, comprehensive data were collected through questionnaires, physical assessments, and sample analyses. The Townsend deprivation index measures regional socioeconomic status, with higher values indicating greater deprivation. A healthy diet score (0–10) was constructed based on intakes of ten food groups, with scores ≥5 indicating a healthy diet [2]. Body mass index was derived by dividing body weight (kg) by the square of height (m^2^). Participants were considered to have adequate physical activity if they engaged in ≥150 minutes of moderate-intensity exercise weekly, ≥75 minutes of vigorous-intensity exercise weekly, or an equivalent combination of the two. Estimated glomerular filtration rate was calculated according to the 2021 Chronic Kidney Disease Epidemiology Collaboration creatinine equation [3]. Medication use for hypertension and dyslipidemia was self-reported. Hypertension history was assessed via self-report, systolic/diastolic blood pressure ≥140/90 mmHg, medical records, or use of antihypertensive medications. Dyslipidemia history was determined based on self-report, triglyceride ≥1.7 mmol/L, use of lipid-lowering medications, high-density lipoprotein cholesterol <1.03 mmol/L for men or <1.29 mmol/L for women, or medical records.

**Differential expression - sliding window analysis**

We employed the differential expression - sliding window analysis to capture plasma protein waves during metabolic aging [4]. Metabolic age ranged from 40 to 70 years, and 31 metabolic age centers were selected, each allowing a ± 1.5-year window. Differentially expressed proteins were identified using the linear regression model:

$$\text{Protein levels = }\text{α}\text{ + }\text{β}_{\text{1}}\text{ × }\text{MA}_{\text{low/high}}\text{ + }\text{β}_{\text{x}}\text{ × x + }\text{ε}$$

where α is the intercept, ε is the residual error, *β*s represent regression coefficients, and x denotes the adjusted covariates, including age, sex, self-reported race, assessment center of participants, Townsend deprivation index, education attainment, body mass index, healthy diet, alcohol consumption, physical activity, smoking status, and estimated glomerular filtration rate.

Protein levels were modeled as dependent variables in multivariable linear regression models, with metabolic age as the independent variable and above-mentioned covariates adjusted. For example, at metabolic age 50 years, metabolic age was dichotomized into two levels: high (50 to 51.5 years) and low (48.5 to 50 years), and the analytic sample was restricted to individuals with a metabolic age of 48.5 to 51.5 years. For each protein, the *P* value of *β*_1_ was obtained and adjusted for multiple testing. Proteins with Bonferroni-corrected *P* <0.05 were considered statistically significant.

**Reference**

1. Levine ME, Lu AT, Quach A, Chen BH, Assimes TL, Bandinelli S, Hou L, Baccarelli AA, Stewart JD, Li Y, Whitsel EA, Wilson JG, Reiner AP, Aviv A, Lohman K, Liu Y, Ferrucci L, Horvath S. An epigenetic biomarker of aging for lifespan and healthspan. Aging (Albany NY). 2018 Apr 18;10(4):573-591. doi: 10.18632/aging.101414. PMID: 29676998; PMCID: PMC5940111.

2. Said MA, Verweij N, van der Harst P. Associations of Combined Genetic and Lifestyle Risks With Incident Cardiovascular Disease and Diabetes in the UK Biobank Study. JAMA Cardiol. 2018;3(8):693-702.

3. Inker LA, Eneanya ND, Coresh J, Tighiouart H, Wang D, Sang Y, Crews DC, Doria A, Estrella MM, Froissart M, et al. New Creatinine- and Cystatin C-Based Equations to Estimate GFR without Race. N Engl J Med. 2021;385(19):1737-1749.

4. Lehallier B, Gate D, Schaum N, Nanasi T, Lee SE, Yousef H, Moran Losada P, Berdnik D, Keller A, Verghese J, et al. Undulating changes in human plasma proteome profiles across the lifespan. Nat Med. 2019;25(12):1843-1850.


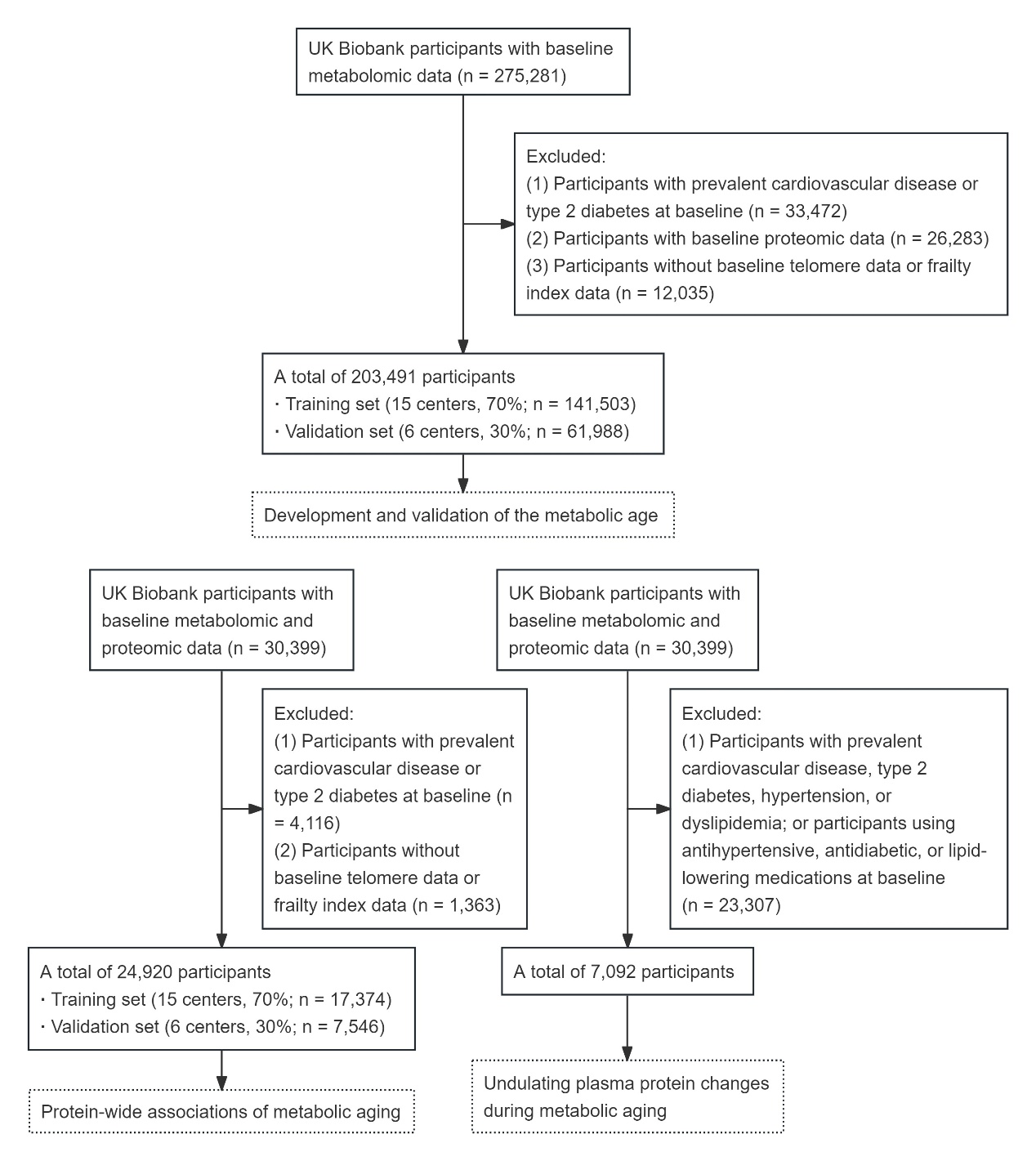


**Fig. S1.** Study flow chart

**
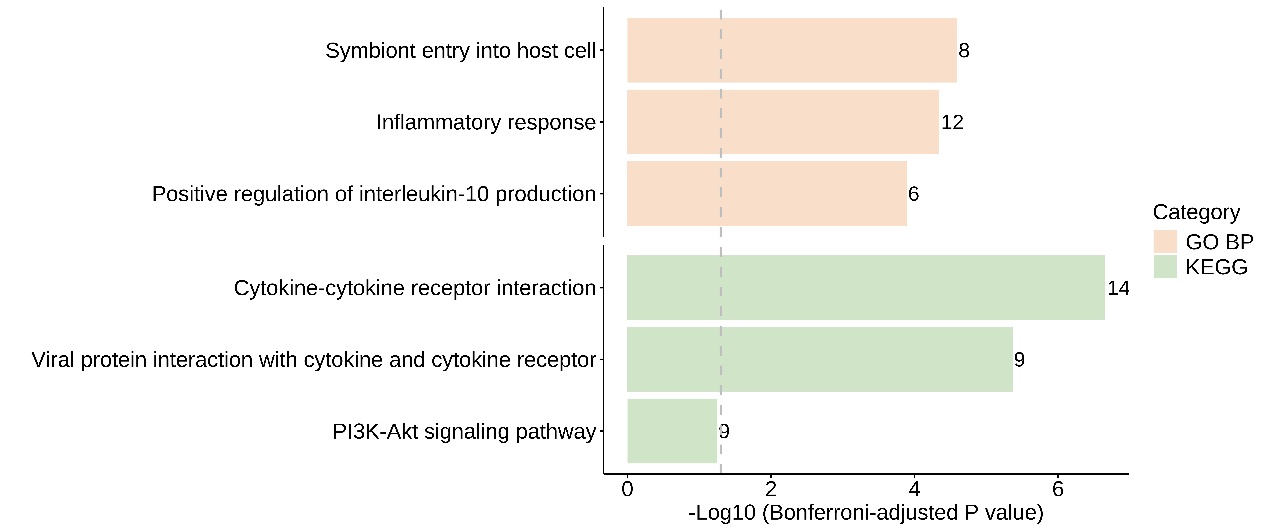
Fig. S2.** Top three enriched pathways of proteins associated with metabolic aging. *P* values were calculated under two-sided tests and statistical significance was defined as a Bonferroni-adjusted *P* <0.05 (dashed vertical line). The number near each bar is the number of observed proteins in each pathway. GO BP, Gene Ontology - biological process; KEGG, Kyoto Encyclopedia of Genes and Genomes.


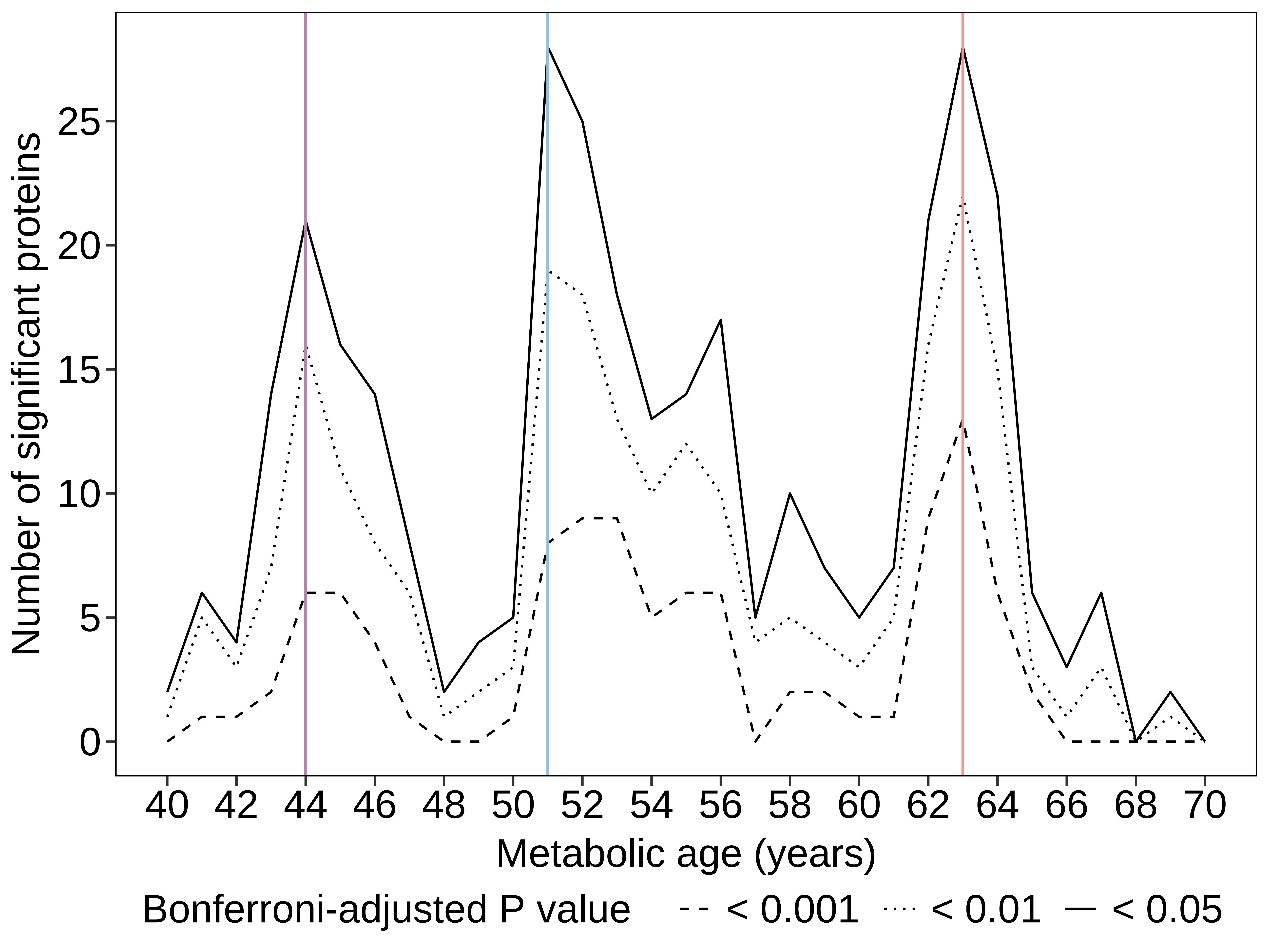


**Fig. S3.** Plasma protein waves during metabolic aging under different thresholds of *P* values.


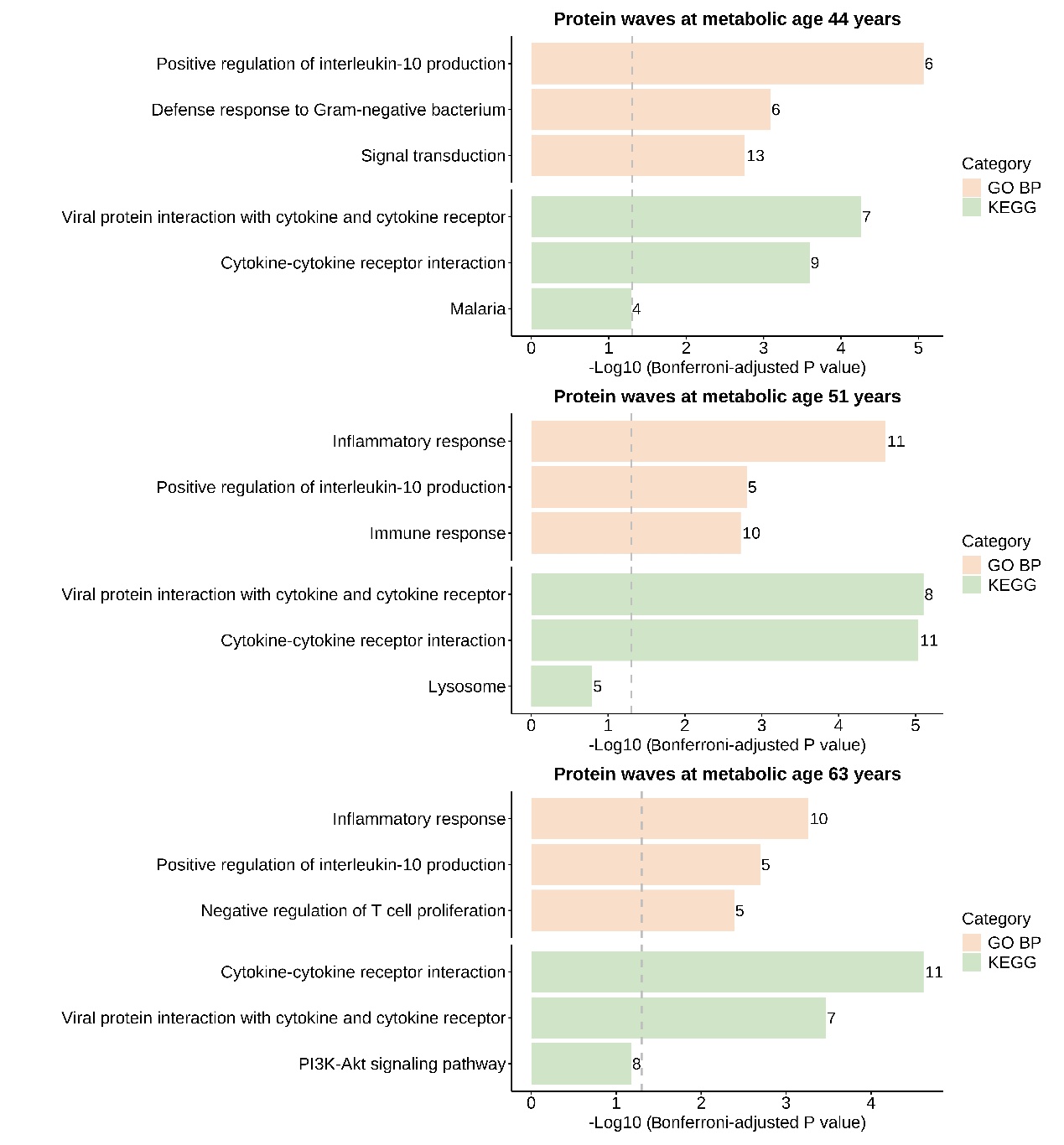
**Fig. S4.** Top three enriched pathways of FDR-significant (*P* <0.05) proteins at different peaks of metabolic age. *P* values in the figure were calculated under two-sided tests and statistical significance was defined as a Bonferroni-adjusted *P* <0.05 (dashed vertical line). The number near each bar is the number of observed proteins in each pathway. FDR, false discovery rate; GO BP, Gene Ontology - biological process; KEGG, Kyoto Encyclopedia of Genes and Genomes.
